# Current Strategies and Future Perspectives for Precision Medicine in Pancreatic Cancer

**DOI:** 10.3390/cancers12041024

**Published:** 2020-04-21

**Authors:** Ivonne Regel, Julia Mayerle, Mahajan Ujjwal Mukund

**Affiliations:** Department of Medicine II, University Hospital, LMU Munich, 81377 Munich, Germany; ivonne.regel@med.uni-muenchen.de (I.R.); ujjwal_mukund.mahajan@med.uni-muenchen.de (M.U.M.)

**Keywords:** pancreatic ductal adenocarcinoma, cancer subtypes, targeted therapy, biomarker, epigenetic, metabolome, PDAC initiatives

## Abstract

Current standard-of-care for patients with pancreatic ductal adenocarcinoma (PDAC) focusses on chemotherapeutic regimens and pancreatic cancer surgery. However, limited treatment options, late diagnosis in advanced tumor stages and the aggressive behavior of PDAC contribute to the high mortality of the disease. Consequently, there is an urgent need of precision medicine for pancreatic cancer patients. All over the world, numerous initiatives started in recent years to translate novel scientific discoveries into prospective clinical trials. One major approach pursues the stratification of PDAC patients according the tumor transcriptome to predict treatment response. Other strategies concentrate on genomic alterations and the identification of individualized targeted therapies. Further experimental studies are ongoing to detect novel biomarkers for cancer diagnosis, subtyping, treatment response prediction or clinical outcome. However, the challenge remains to transfer the knowledge into clinical practice. In this review, we summarize current literature and knowledge and highlight novel concepts of basic and clinical research uncovering suitable biomarkers and targeted therapies. Thus, we provide an overview of preclinical and clinical efforts of precision medicine in pancreatic cancer.

## 1. Introduction

Pancreatic ductal adenocarcinoma (PDAC) is the most common type of cancer in the pancreas and is burdened with a poor prognosis. Although the five-year overall survival increased from 5% to 9% over the past decade [1], the malignant disease still has a dismal outcome. The small progress was mainly achieved through recent improvements in neoadjuvant and adjuvant therapeutic strategies as well as perioperative care [2]. However, therapeutic options are still limited and the tumor often develops resistance to current treatment modalities. The relevant standard-of-care treatment options for resectable tumors and locally advanced or metastasized PDAC are summarized here [2,3]. Based on the recently published results from the PRODIGE 24-PA6 (NCT01526135) trial, adjuvant standard treatment after tumor resection changed from gemcitabine to modified FOLFIRINOX (folinic acid, fluorouracil, irinotecan and oxaliplatin), in patients with good performance status. The median disease-free survival (DFS) was reported with 21.6 months in the FOLFIRINOX group compared to 12.8 months in the gemcitabine group [4]. According to posted study results of the APACT (NCT01964430) trial on clinicaltrials.gov, there was no significant benefit in DFS (updated version 6 January 2020) for gemcitabine plus nab-paclitaxel versus gemcitabine alone in the adjuvant setting of PDAC treatment. (https://clinicaltrials.gov/ct2/show/results/NCT01964430?view=results).

Although there is notable progress in resectable PDAC cancer and adjuvant chemotherapy, we are far away from a cure, since the recurrence rate is 80–90% after five years. Moreover, only 10–20% of pancreatic cancer patients are suitable for surgical resection [5]. Unfortunately, the majority of PDAC patients are diagnosed at late tumor stages with locally advanced or metastatic pancreatic cancer. Here, the standard-of-care also focusses on FOLFIRINOX or gemcitabine-based therapies and the median overall survival time with systemic chemotherapy ranges from 6–18 months for patients with advanced or metastatic pancreatic cancer [2]. Notably, the current clinical data reveal that chemotherapeutic approaches alone are probably not capable of increasing response rates and survival time in the near future. Thus, clinicians and researchers are placing high hopes on precision medicine and the identification of suitable biomarkers and targeted therapies.

## 2. Selected Mutant Gene Profiles and Cancer Subtypes are Eligible for Targeted Therapy

The mutational landscape of PDAC is in comparison to other tumor entities quite homogeneous [6]. Several studies identified four genes that are predominantly affected in PDAC patient samples: *KRAS* (Kirsten rat sarcoma viral oncogene homolog), *CDKN2A* (cyclin-dependent kinase inhibitor 2A, p16), *TP53* (tumor protein 53) and *SMAD4* [7,8,9,10]. The tumor suppressor genes *CDKN2A*, *TP53* and *SMAD4* are mostly inactivated through genomic mutations, although *CDKN2A* can also be silenced through homozygous deletions or DNA methylation [11]. In contrast, mutations in the oncogene *KRAS* lead to a constitutive activation of the RAS signaling pathway. A missense mutation, mostly located in one of the three hot-spots (G12, G13 and Q61), is responsible for the inhibition of the GTPase activity, which retains KRAS in its active GTP-bound form [12]. Until recently, it was assumed that mutant KRAS is not a suitable target for cancer therapy. However, the identification of a new small molecule AMG 510, showing a high potency and efficiency in inhibiting KRAS(G12C)-mediated signaling, is expected to be a great success. The first results of the study displayed tumor regression in AMG 510-treated xenograft mouse models. Moreover, in a preliminary clinical evaluation (NCT03600883), four patients with KRAS(G12C)-mutant non-small-cell lung cancer (NSCLC), who received the inhibitor constantly, demonstrated either partial response or stable disease, demonstrating a great treatment efficacy [13]. Although the KRAS(G12C) mutation is very rare in PDAC patients [12], it might be a great opportunity for a personalized therapy in a subgroup of patients. 

**Figure 1:**
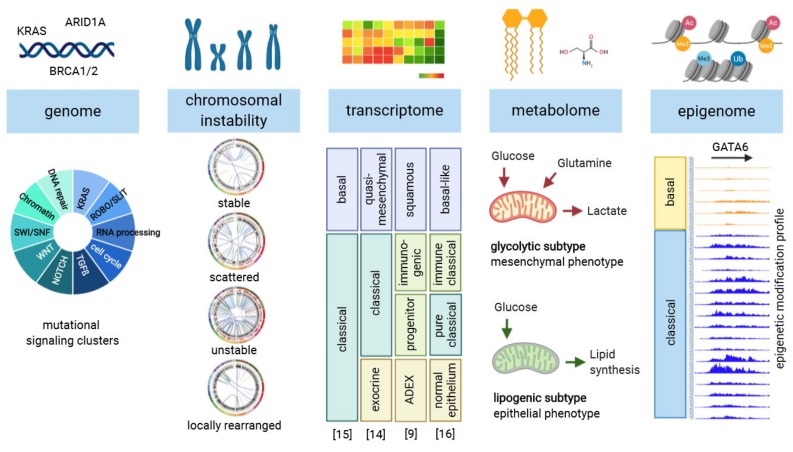
Subtyping of pancreatic ductal adenocarcinoma based on various molecular tumor features. Genome wide mutations, detected in pancreatic ductal adenocarcinoma (PDAC) tissue, cluster in specific pathways that are associated to cancer formation [9]. Analysis of chromosomal instability and structural rearrangements revealed four different PDAC subtypes [10]. Transcriptomic cluster analysis shows a variation in PDAC subtypes when compared in different studies [9,14,15,16]. Metabolome analysis of PDAC displays a separation into a glycolytic and lipogenic tumor subtype [17]. The epigenomic landscape of PDAC tissue reflects the classical and basal transcriptomic subtypes, data adapted from [18].

Whole-exome and -genome sequencing approaches detected, besides the main driver mutations, other mutated genes with a lower frequency (≤10%) in PDAC patients [7,8,9,10]. The mutated genes can be further grouped into tumor-related processes and pathways that affect tumor cell behavior (Figure 1). Targeted treatment addressing the dysregulated pathways in individual patients is not easy to accomplish since the genetically altered pathway components show a great variety from patient to patient [7,9]. A great set of mutated genes coding for epigenetic remodeling enzymes were detected in ~35% (134/383) of PDAC patients, although, single genes show a mutation frequency of only 0.5% to 8% (TCGA dataset, Pancreatic Adenocarcinoma, QCMG, Nature 2016) [9]. For details, see Table 1. As long as we do not identify common regulatory mechanisms, which are suitable for therapeutic targeting, we have to address the genomic complexity of each patient individually to assign the best treatment option.

Besides gene mutation profiles, PDACs can be additionally sub-classified according their chromosomal stability in a stable, locally rearranged, scattered and unstable subtype (Figure 1). Here, gene disruptions, gene amplifications (copy number alterations) or oncogene fusions can occur through chromosomal rearrangement. Interestingly, the unstable tumor subtype is associated with defects in the DNA-damage response (DDR) pathway and shows a high abundance of BRCA mutations [10]. Overall, mutations, either germline or somatic, in the DDR pathway were identified in 9–15% of PDAC patient samples, whereby *ATM* and *BRCA2* mutations were with 4–4.5% and 2.1–2.9% most frequent [10,19]. For details, see Table 1. Pishvaian and colleagues categorized them, among others, as “actionable” mutations for which a targeted therapy is available [19]. More than a decade ago, it was shown that cells deficient for BRCA1 and/or BRCA2 are sensitive towards poly (ADP-ribose) polymerase inhibitors (PARPi) [20,21]. PARP is a DNA damage sensor and its inhibition induce DNA double strand breaks. Hence, tumor cells lacking the DNA double-strand break repair enzymes BRCA1 and BRCA2 are unable to repair DNA damages and undergo cell death. Notably, a platinum-based chemotherapy also causes DNA damage so that DNA repair deficient tumors show increased treatment sensitivity [22]. A randomized phase 3 trial (POLO, NCT02184195) enrolled metastasized PDAC patients with a germline *BRCA1* or *BRCA2* mutation whose cancer had not progressed under first-line platinum-based therapy. Patients receiving the PARPi olaparib as a maintenance therapy displayed significantly longer progression-free survival compared to the placebo group. However, the interims analysis showed no difference in overall survival rate. Moreover, around 22% of BRCA-mutant patients showed disease progression under platinum-based therapy and were therefore not eligible for the trial [23]. Consequently, a more precise profiling of BRCA-mutant patients is required to identify patients who will benefit from a PARPi or platinum-analogue treatment. Further strategies of targeting actionable mutations are ongoing (see also review from Nevala-Plagemann [24]); a prospective proof of effectiveness and performance for biomarker-selected therapies remains to be confirmed in pancreatic cancer patients.

Another approach for stratifying pancreatic cancer patients utilizes transcriptomic changes of the tumor tissue (Figure 1). Different research groups identified pancreatic cancer subclasses based on alterations in gene expression that are associated with tumor grade, survival rate and therapy response. The study of Collisson et al. introduced in 2011 the classical, quasi-mesenchymal and exocrine PDAC subtype. They provided a gene signature for each subtype and demonstrated that the quasi-mesenchymal type has the worst prognosis but shows increased sensitivity to gemcitabine treatment [14]. The molecular profiling of Moffitt et al. in 2015 distinguished between a classical and basal-like PDAC subtype and they detected a significantly decreased overall survival for the basal-like subtype. In addition, they virtually separated the transcriptome of the fibroinflammatory compartment from the tumor and identified a silent and activated stroma signature. Although the stroma signature was independent from the PDAC subtypes, patients with active stroma exhibited a worse prognosis [15]. In 2016, Bailey et al. defined the squamous, ADEX (aberrantly differentiated exocrine), pancreatic progenitor and immunogenic PDAC subtypes, showing prognostic relevance. The squamous and ADEX subtype directly overlap with the quasi-mesenchymal and exocrine subclasses of Collisson et al., whereby the classical subtype was sub-classified in a pancreatic progenitor and immunogenic subtype [9,14]. Overall, the PDAC subtypes are highly consistent, and a combined molecular profiling displayed a prominent separation into two subclasses consisting of a classical/pancreatic progenitor and a quasi-mesenchymal/basal-like/squamous subtype. A further subdivision into an exocrine/ADEX or immunogenic subtype highly depends on the stroma composition [16,25]. Ex-vivo cultivation of isolated PDAC cells as organoid cultures has demonstrated a similar subtyping into a classical and basal-like phenotype. Moreover, the individual tumor organoids were utilized for a pharmacotyping to guide treatment decisions [26]. Although huge progress was made in defining molecular PDAC subtypes with a prognostic value, their impact on treatment decisions still needs to be evaluated. However, cancer subtyping, based on genetic alterations as in the DDR pathway or based on transcriptomic signatures, has a great chance for clinical translation. Several worldwide initiatives focus on how to implement the stratification approaches in personalized treatment decisions and clinical management of PDAC patients. 

## 3. Precision Medicine Initiatives

The precision medicine initiatives aim at identifying an individual effective therapy for a cancer patient. Therefore, genetic, molecular, environmental and lifestyle factors of cancer patients are considered to provide the best treatment opportunity. In contrast to a conventional therapy of a specific cancer entity, precision medicine considers individual patient differences and stratifies patients accordingly. Defining a concept of precision medicine for pancreatic cancer patients has become quite popular in recent years. As described above, many outstanding work groups have made tremendous efforts to uncover genetic and molecular alterations in pancreatic cancer patients. They provided fundamental work to stratify patients in distinct subclasses exhibiting prognostic relevance or possible treatment advantages, also reviewed in [27]. However, the overall patient benefit of the classification remains to be established in prospective clinical trials. The implementation of precision medicine approaches into clinical practice is a huge challenge for future years. Different initiatives were founded all over the world to determine tumor biology characteristics and to offer the best treatment options for pancreatic cancer patients. The Pancreatic Cancer Action Network (PanCAN) in the United States (https://www.pancan.org/) runs a precision medicine program, called Know Your Tumor^®^, where a personalized treatment strategy is suggested based on a molecular tumor profiling [19]. Initial results from the program revealed that 50% of pancreatic cancer patients have an “actionable” molecular alteration for which targeted therapy is available. A small patient cohort changed from standard-of-care treatment to matched molecular-targeted therapy exhibited a significantly improved progression-free survival, after a correction for the line of therapy [19]. However, more prospective studies are needed to address the efficacy of targeted agents in biomarker-driven pancreatic cancer therapy. Thus, the PanCAN initiative started Precision Promise^SM^ (https://www.pancan.org/research/precision-promise/), a response-adaptive clinical trial platform with multiple treatment arms testing simultaneously various therapeutic opportunities. In the United Kingdom, the pancreatic cancer initiative Precision-Panc (https://www.precisionpanc.org/) pursues a similar approach. Each registered patient in Precision-Panc receives a genomic profiling and molecular phenotyping utilizing next-generation sequencing and RNA sequencing, respectively [28]. Under the guise of PRIMUS (Pancreatic canceR Individualised Multi-arm Umbrella Studies), different clinical studies, addressing treatment strategies and/or biomarker development for prognosis or treatment response, are enrolled to offer precision medicine clinical trials to pancreatic cancer patients [29]. Some of the studies contain an integrated biomarker evaluation concept to determine if a biomarker positive group shows a better treatment performance. So far, the biomarker-driven approaches concentrate on molecular markers predicting a better treatment response towards gemcitabine or FOLFIRINOX. The Canadian initiative EPPIC, enhanced pancreatic cancer profiling for individualized care (https://www.tfri.ca), launched the COMPASS trial, which was the first prospective translational study for advanced PDAC integrating PDAC subtypes and chemotherapy response. Although the classical PDAC subtype shows a better outcome after FOLFIRINOX treatment, there is no alternative standard treatment option for the basal-like subtype, given that FOLFIRINOX should be preferred if the patient shows a good performance status [30]. Notably, similar clinical initiatives studying different aspects of patients diagnosed with PDAC are initiated in Germany. Thus, a collaborative project named PREDICT-PACA (Integrated Biopsy-Based Approach to Predict Response to Chemotherapy for Patients with Stage IV Pancreatic Cancer) will start in 2020, addressing molecular and biological tumor features to predict chemotherapy response. In addition, two prospective clinical studies, ESPAC-6 and ESPAC-7, are planned to evaluate oxaliplatin- or gemcitabine-based chemotherapy response of PDAC patients that will be randomized according standard clinical criteria or by transcriptomic stratification signatures [26]. These concepts will increase the availability of precision medicine approaches to PDAC patients in Germany and Europe. To improve the outcome of PDAC patients, the Australian Pancreatic Cancer Genome Initiative (APGI, https://www.pancreaticcancer.net.au/) concentrates on genomic and epigenomic alterations in tumor tissue. In 2015, they published the IMPaCT (Individualized Molecular Pancreatic Cancer Therapy) trial demonstrating the feasibility of collecting and screening biomaterial for actionable molecular targets. In addition, the APGI consortium genetically classified one of the largest PDAC cohort into four molecular tumor subtypes, which we described above [9]. All these different initiatives comprise comprehensive biobanks annotated with clinical and genomic data. They hope to improve the understanding of individual tumor biology to detect new biomarkers and facilitate personalized treatment strategies. 

## 4. Novel Preclinical Strategies for Precision Medicine

Current clinical efforts mainly focus on the therapeutic relevance of genomic and molecular subtypes. However, a restriction on these two levels might be of limited value considering the complex nature of pancreatic cancer. Changes in the epigenome, proteome and metabolome highly contribute to phenotypical alterations in pancreatic cancer and play a major role in the stratification of PDAC patients [17,18,31]. In a proteome-based analysis on PDAC liver metastases, Law and colleagues uncovered metabolic, progenitor-like, proliferative and inflammatory PDAC subtypes with a high concordance to the transcriptomic subclasses [9,14,15,31]. Notably, the classification of an inflammatory cancer subtype emphasizes the importance of the tumor microenvironment affecting PDAC characteristics. The substantial and complex impact of the inflammatory tumor microenvironment on tumor biology and therapy efficiency is excellently reviewed in [32,33]. In a previous study, we could show that the immune microenvironment in PDAC can be of prognostic value and we provided a signature comprising different leukocyte subpopulations and stromal composition to stratify patients using progression free survival as the primary outcome [34]. Similarly, a study of Wartenberg et al. identified three distinct immune subtypes of PDAC that were associated with patient prognosis [35]. The discovery of predictive biomarkers for responders of immunotherapies would be a major achievement in the future. Many preclinical studies are ongoing to identify new biomarkers for cancer diagnosis, subtyping, treatment response prediction or clinical outcome; however, the challenge remains to transfer the knowledge into clinical practice. In the following, we give an overview on preclinical efforts addressing metabolic and epigenetic mechanisms for precision medicine in pancreatic cancer. 

### 4.1. Opportunities of Metabolic Profiles to Develop Targeted Therapies

The ultimate goal of most metabolomic cancer studies is to discover cancer-specific diagnostic, prognostic or predictive biomarkers for an individual patient [36]. The National Institutes of Health Biomarkers Definitions Working Group defined a biomarker as “a characteristic that is measured as an indicator of normal biological processes, pathogenic processes, or responses to exposure or intervention, including therapeutic interventions” [37]. An ideal biomarker should meet various criteria that include, (i) it should be present in readily available and minimally invasive sources (e.g., blood and urine); (ii) it should be highly sensitive (allowing early diagnosis) and specific (unaffected by external and comorbid conditions); (iii) it should vary promptly in response to treatment and disease progression; (iv) it should provide a deeper understanding about the disease mechanism; and (v) it should be useful in risk stratification and prognosis [38]. Metabolomics has an advantage over other “-omics” and is better suited for this purpose. In fact, as changes in metabolites normally appear in readily available biofluids, such as blood and urine, the translation of metabolomic studies to clinical practice is easier [39]. Biofluids are usually the easiest samples to work with, requiring less sample preparation than other biological samples [40]. The metabolome is highly dynamic, reflecting continuous fluxes of both metabolic and signaling pathways, and is sensitive to diverse host and environmental factors. These unique features make metabolomics able to capture a plurality of subtle changes. Thus, metabolomics holds the promise for simultaneously evaluating a variety of complex pathways and their consequences [39]. Moreover, metabolomics experiments are also less expensive than proteomic and transcriptomic approaches [36,40,41,42]. These biomarkers can, in turn, be used to develop personalized prognostic, diagnostic, and treatment approaches, and can also be applied to monitor disease progression, treatment efficacy, predisposition to drug-related side effects, and potential relapse [43].

Although targeting cancer metabolism is a promising therapeutic strategy, clinical success will depend on accurate diagnostic identification of tumor subtypes with specific metabolic requirements. Through broad metabolite profiling, PDAC was successfully categorized into three highly distinct metabolic subtypes, namely slow proliferating, glycolytic and lipogenic subtype [17]. One subtype was defined by reduced proliferative capacity, whereas glycolytic and lipogenic subtypes showed distinct metabolite levels associated with glycolysis, lipogenesis, and redox pathways. The lipogenic subtype associated with an epithelial phenotype, whereas the glycolytic subtype strongly associated with a mesenchymal subtype, suggesting functional relevance in disease progression (Figure 1) [17,44]. This identification of distinct metabolic subtypes in PDAC may inform patient selection for investigational metabolic inhibitors and in the selection of new therapeutic targets [17,45]. The only routinely used serum marker for PDAC patients with demonstrated clinical usefulness in therapeutic monitoring and early detection of recurrent disease after treatment is carbohydrate antigen 19-9 (CA 19-9) [46]. Elevation of CA 19-9 signifies advanced PDAC and poor prognosis [47,48]. However, the elevation of CA 19-9 is observed in only 65% of patients with resectable PDAC [47,49] and can also be caused by other conditions such as pancreatitis, cirrhosis and cholestasis [50]. In addition, patients who are negative for Lewis antigen a or b (approximately 10% of patients with PDAC) are unable to synthesize CA 19-9 even in advanced stages of the disease. Although measurement of serum CA 19-9 levels is useful in patients with known pancreatic cancer, the application of this biomarker as a screening tool revealed disappointing results and is not recommended as a screening marker for PDAC [46,47,51]. Our working group recently identified a metabolite-based biomarker signature, which distinguishes PDAC from CP with much greater accuracy than CA 19-9 alone [52]. Mayerle et al. performed in 914 patients a global analysis of blood metabolites, including lipids, to uncover candidate metabolites that distinguish PDAC from CP. Notably, from 477 metabolites out of 10 ontology classes, 29 metabolites were significantly altered between PDAC and CP in serum and plasma samples of the training set. Finally, a nine-metabolite signature plus the CA 19-9 model discriminated PDAC from CP with AUC of 0.96, and could be successfully validated in an independent cohort [52]. The study was designed to accurately exclude suspected pancreatic cancer in CP patients, with emphasis placed on optimizing the negative predictive value (NPV) [52,53]. In another metabolomic approach, Phua et al. established metabotyping of resected PDAC tissue to predict gemcitabine treatment response in an adjuvant setting [54]. This proof-of-principle work showed that distinct tissue metabolomes, consisting of various amino acids, fatty acids, nucleobase intermediates and inorganic acids, could be used to depict the treatment response of gemcitabine [54]. The near future probably lies in a carefully selected panel of metabolome biomarkers that would allow for earlier diagnosis of PDAC, an easier determination of its stage and, ideally, provide indicators of prognosis or outcome.

### 4.2. Epigenetic Therapies for Defined Subtypes

The epigenetic landscape determines cell type-specific molecular profiles through distinct DNA methylation and histone modification patterns at regulatory genomic regions. Gene silencing is, for example, associated with DNA hypermethylation at promoter regions, or the trimethylation of histone 3 on lysine 27 (H3K27me3) and/or the mono-ubiquitination of histone 2A at lysine 119 at various gene regulatory elements. In contrast, an enrichment of histone acetylation and/or the trimethylation of lysine 4 on histone H3 (H3K4me3) are associated with gene activation [55]. Interestingly, epigenetic mechanisms are able to implement external or internal stimuli into gene expression signatures affecting cellular phenotypes, without changing the underlying DNA sequence. Since genetic variants cannot fully explain the different subtypes of pancreatic cancer, a consideration of the epigenetic status in PDAC tumor tissues could be relevant for therapy response prediction and clinical outcome. Moreover, the reversible nature of epigenetic modifications and a reprogramming of the epigenetic landscape harbors a great potential for pancreatic cancer treatment. In a recent study, we have shown that a comprehensive epigenetic remodeling process, accompanied by an epigenetic silencing of differentiation genes, is a mandatory event in pancreatic cancer development [56]. Our data indicate that a defined epigenetic gene signature might predict the likelihood of malignant transformation. In addition, we have demonstrated that the depletion or therapeutic inhibition of the epigenetic remodeler Ring1b reprograms pancreatic cancer cells towards a less aggressive tumor phenotype [56]. Although many epidrugs (drugs, which target the epigenome) show striking results in preclinical studies, their effectiveness in clinical trials for solid cancer is rather disappointing [57]. A benefit for PDAC patients might depend on epigenetic cancer signatures, pre-classifying patients eligible for an epigenetic therapy. An emerging set of pre-clinical studies were conducted to investigate epigenetic profiles in pancreatic cancer, which reveal a correlation to pancreatic cancer phenotypes and their characteristics. Besides the increased expression of an epigenetic remodeler, many oncogenic pathways are epigenetically regulated, demonstrating an important role of epigenetic mechanisms in PDAC formation [18,56]. Moreover, a cluster analysis based on chromatin states revealed two distinct patterns (Figure 1), mainly resembling the classical and basal transcriptional subtype as previously identified by Moffitt et al. [15,18]. Particularly, histone acetylation profiles at distal regulatory genome regions have a high informative value and contribute to phenotypic variations in high- and low-grade PDAC [58]. However, targeting histone acetylation patterns by either histone deacetylase (HDAC) or histone acetyltransferases (HAT) inhibitors is a non-selective process resulting in global increased or decreased histone acetylation levels, respectively. In contrast to the classical subtype, the basal-like subtype demonstrates a loss of histone acetylation at differentiation genes [18]. Consequently, HDAC inhibitor treatment of basal-like PDAC cells might result in a gain of histone acetylation at differentiation genes and a reprogramming towards a less aggressive phenotype. Thus, the characterization of histone acetylation patterns on classifier genes might be of great importance to predict the epigenetic reprogramming efficiency of tumor cells. Importantly, the application of an epigenetic therapy requires, as any other targeted therapy, a prior selection of eligible patients. Without a stratification of PDAC patients, based on transcriptome or epigenetic signatures, further clinical trials will most likely fail, although an epigenetic therapy might reveal a great chance for a subgroup of cancer patients.

## 5. Conclusions and Perspectives

In recent years, we collected increasing knowledge of genetic and molecular alterations in pancreatic cancer. Tremendous efforts were conducted to uncover various PDAC subtypes and actionable mutations aiming for targeted therapies. Consequently, precision medicine initiatives started to translate scientific discoveries into clinical management to improve the dismal prognosis. A comprehensive prospective evaluation of proposed patient stratification systems is urgently needed. So far, it has not been possible to translate promising pre-clinical findings, such as PDAC subtypes or whole genome profiles, into a true patient benefit. One major challenge is to evaluate basic science results in clinical settings or to select the most encouraging scientific data for conducting a clinical trial. A close collaboration of basic and clinician scientists is of great importance to overcome these hurdles.

## Figures and Tables

**Table 1:** Mutation frequency of coherent pathway components in PDAC patients. Data according The Cancer Genome Atlas (TCGA) dataset pancreatic adenocarcinoma (QCMG, Nature 2016) with genomic information for 383 PDAC patients [9]. DDR, DNA damage response.

Pathway	Incidence Multiple Genes		Incidence Single Genes	
**Epigenetic regulators**	~35% (134/383)	*ARID1A, KMT2C, KMT2D, KDM6A, SMARCA4, SETD2, ARID2, PBRM1, HDAC1, CREBBP, SETDB1, SETD1B, EP300, JARID2, KMT2A, SMARCA1, SMARCA2, KDM5C, SETBP1, KDM2B, ARID3C, DNMT3B, DNMT1, ARID4A, KDM5A, SMARCB1, SMARCD1, SETD1A, KAT6B*	8%	*ARID1A*
			5%	*KMT2C*
			2.9%	*SMARCA4*
			2.3%	*SETD2*
**DDR pathway components**	~9% (34/383)	*BRCA1, BRCA2, PALB2, ATM, ATR, MLH1, MSH2, MSH6, RPA1, STK11, FANCA, FANCC*	4%	*ATM*
			2.1%	*BRCA2*
			1.3%	*BRCA1*
			0.5%	*PALB2*
